# NORAD01-GRECCAR16 multicenter phase III non-inferiority randomized trial comparing preoperative modified FOLFIRINOX without irradiation to radiochemotherapy for resectable locally advanced rectal cancer (intergroup FRENCH-GRECCAR- PRODIGE trial)

**DOI:** 10.1186/s12885-020-06968-1

**Published:** 2020-05-29

**Authors:** Antoine Brouquet, Jean-Baptiste Bachet, Florence Huguet, Mehdi Karoui, Pascal Artru, Charles Sabbagh, Jérémie H. Lefèvre, Dewi Vernerey, Christophe Mariette, Eric Vicaut, Stephane Benoist, on behalf the FRENCH , GRECCAR, PRODIGE study groups

**Affiliations:** 1 Service de Chirurgie Digestive et Oncologique, Hôpital Bicêtre, Groupe Hospitalier Universitaire Paris Sud, Assistance Publique, Hôpitaux de Paris, 63, rue Gabriel Péri, Le Kremlin Bicetre, 94275 France; 2 Faculté de Médecine Paris Sud, Université Paris Saclay, Lrekmlin Bicêtre, 94275 France; 3 Service d’Oncologie Digestive, Hôpital de la Pitié Salpétrière, Assistance Publique, Hôpitaux de Paris, Paris, 75013 France; 4 Service de Radiothérapie, Hôpital Tenon, Assistance Publique, Hôpitaux de Paris, Paris, 75020 France; 5 Service de Chirurgie Digestive, Hôpital Européen Georges Pompidou, Assistance Publique, Hôpitaux de Paris, Paris, 75015 France; 6 Centre d’oncologie Jean Mermoz, Lyon, France; 7 Service de Chirurgie Digestive, CHU Amiens, Amiens, 60000 France; 8 Service de Chirurgie Générale et Digestive, Hôpital Saint Antoine, Assistance Publique, Hôpitaux de Paris, Paris, 75012 France; 9 Unité de recherche clinique CHU, Besançon, France; 10 Service de Chirurgie Digestive et Oncologique, CHU Lille, Lille, 59000 France; 11 Unité de Recherche Clinique Paris VII, Assistance Publique, Hôpitaux de Paris, Paris, 75010 France

**Keywords:** Locally advanced rectal cancer, Circumferential resection margin, Preoperative chemotherapy, Preoperative radiochemotherapy, Quality of life, Functional result

## Abstract

**Background:**

Preoperative radiochemotherapy (RCT) is recommended in France prior to total mesorectal excision in patients with mid or low locally advanced rectal cancer (LARC) (cT3/T4 and/or N+) because it has been shown to improve local control. Preoperative RCT has also disadvantages including the absence of proven impact on metastatic recurrence and the risk of late side effects on bowel and genitourinary function. In patients with primarily resectable LARC, preoperative systemic chemotherapy without pelvic irradiation could be used as an alternative to RCT.

**Methods:**

This study is a multicenter, open-label randomized, 2-arm phase III non-inferiority trial. Patients with mid or low resectable LARC (cT3N0 or cT1-T3N+ with circumferential resection margin [CRM] > 2 mm on pretreatment MRI) will be randomized to either modified FOLFIRINOX for 3 months or RCT (Cap50 intensified-modulated radiotherapy). All patients have restaging MRI after preoperative treatment. The primary endpoint is 3-year progression-free survival (PFS) from the time to randomization including progression during preoperative treatment. Secondary endpoints are treatment related toxicity, treatment compliance, R0 resection rate, sphincter saving surgery rate, postoperative morbidity and mortality rates, loco-regional recurrence free survival, overall survival, bowel and sexual functions at diagnosis, quality of life, radiologic and pathologic response after preoperative treatment. The number of patients required is 574.

**Discussion:**

The choice of modified FOLFIRINOX for preoperative chemotherapy is supported by recent and consistent data on safety and efficacy of this regimen on rectal cancer. The use of preoperative chemotherapy instead of RCT could be associated with pronounced advantages in terms of functional results and quality of life in cancer survivors. However and first of all, the non-inferiority of preoperative chemotherapy compared to RCT on oncologic outcome has to be validated. If this study demonstrates the non-inferiority of chemotherapy compared to RCT, this can lead to a crucial change in clinical practice in a large subset of rectal cancer patients.

**Trial registration:**

ClinicalTrials.gov NCT03875781 (March 15, 2019).

Version 1.1.

## Background

Local control of rectal cancer has been considerably improved by the standardization of the surgical technique of mesorectal excision [1] and by preoperative treatment including or based on radiation therapy [2–4]. The risk of local recurrence in rectal cancer patients undergoing surgery after preoperative radiochemotherapy (RCT) currently ranges from 2.9 to 7.6% in most recent controlled randomized trials [2, 4–6]. Preoperative RCT has therefore been widely admitted as a standard of care in combination with total mesorectal excision in patients with locally advanced middle or low rectal cancer (cT3/T4 and/or N+). However, all patients with locally advanced rectal cancer (LARC) may not benefit from preoperative pelvic irradiation. The risk of local recurrence after rectal cancer surgery is closely linked to circumferential resection margin (CRM) and consistent data have shown that this parameter can be accurately predicted preoperatively on MRI [7, 8]. As such, CRM-based strategies have been proposed to select patients in whom irradiation could be safely omitted [8, 9]. On the other hand or furthermore, preoperative pelvic irradiation has never been clearly shown to reduce the risk of metastatic recurrence and to improve overall survival in patients who underwent rectal cancer surgery [10]. Finally, consistent data have shown that preoperative pelvic irradiation is associated with a higher risk of long term digestive and genitourinary dysfunction compared to patients undergoing surgery alone [11, 12]. A number of recent studies suggest that preoperative full-dose oxaliplatin-based chemotherapy could be efficient for local control and used as an alternative to RCT in selected LARC patients [13–15]. This approach could have the advantage to limit the risk of local recurrence after surgery by analogy to RCT but without the risk of late side effects associated with pelvic irradiation. The administration of preoperative chemotherapy could also have of positive effect on the risk of metastatic recurrence and long-term survival.

## Methods and design

### Study overview

**Fig. 1:**
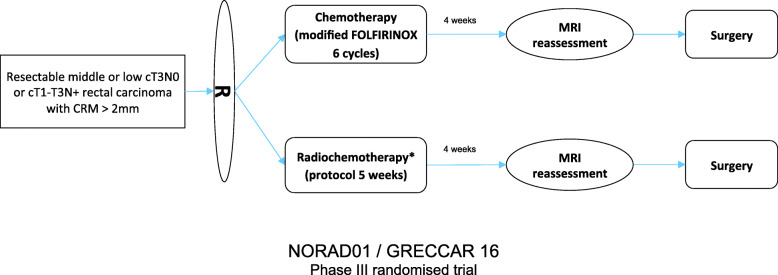
study design

This study is a phase III multicenter randomized open-label controlled trial in parallel groups, comparing preoperative systemic chemotherapy using 6 cycles of modified FOLFIRINOX without pelvic irradiation to standardized RCT in patients with resectable LARC. This study aims to demonstrate the non-inferiority of preoperative chemotherapy using modified FOLFIRINOX without pelvic irradiation compared to RCT on oncologic outcomes in locally advanced resectable low or middle rectal cancer (cT3N0 and/or cT1-T3N+). In this study, resectable rectal cancer is defined by cT3 and/or cN+ rectal tumors with predictive circumferential resection margin > 2 mm can therefore be defined as locally advanced primarily resectable rectal cancer. All patients have pretreatment MRI. The study design is shown in Fig. 1. The primary endpoint is progression-free survival.

### Participants

Patients are included from several departments of surgery or oncology (*n* = 40) in France (see list of participating centers in the Supplementary data 1) after validation of. All participating sites signed a convention with the institutional promoter (Assistance Publique-Hôpitaux de Paris – DRCI: Département de la Recherche Clinique et de l’Innovation, Saint Louis Hospital, 75,475 PARIS) for ethical approval before beginning of inclusion. The study is coordinated by 3 investigators-coordinators (SB, JBB, FH) that have the responsibility of the selection and the validation of the participating centers. The monitoring of the inclusion in the trial and the methodological aspects (collection, management, analysis, and interpretation of data) of the research are under the responsibility of the Unité de Recherche Clinique of Lariboisière Saint-Louis, Assistance Publique Hôpitaux de Paris directed by Pr E. Vicaut. A steering committee including several investigators of the trial (SB, AB, FH, JBB, EV) is responsible for all the decisions that have to be taken regarding the study including the overall organization of the study, coordination of the information, initial methodology and monitor the research process, suggestion of procedures to be followed during the study, acknowledging the recommendations of the Data and Safety Monitoring Board.

### Screening of eligibility criteria

Eligible patients are screened in outpatient consultation by the investigators at each participating centers. All patients are required to have a complete work up of rectal cancer including clinical examination (previous history of colorectal cancer or other neoplasia, physical examination, assessment of WHO/ECOG performance status, assessment of digestive symptoms), complete colonoscopy with biopsy, rectal MRI, CT scan of the chest, the abdomen and the pelvis, and blood sample tests with tumor markers, and serum pregnancy test. Inclusion and exclusion criteria are as follows:

#### Inclusion criteria

For inclusion in the study, all of the following inclusion criteria must be fulfilled: histologically proven middle or low rectal carcinoma, ≤ 10 cm from the anal verge on MRI (sagittal slide), cT3N0 and/or cT1-T3N+ on pretreatment imaging work up (pelvic contrast enhanced MRI and/or endorectal ultrasound), pretreatment predictive circumferential margin > 2 mm on pretreatment imaging work up (pelvic contrast enhanced MRI), patients must be 18 years old or older, a World Health Organization (WHO/ECOG) performance status of 0 or 1, informed consent signed, patients of childbearing / reproductive potential should use adequate birth control measures during the study treatment period and for at least 6 months after the last study treatment. A highly effective method of birth control is defined as those which result in low failure rate (i.e. less than 1% per year) when used consistently and correctly. Presumed node invasion (cN+) is defined as the presence of at least one mesorectal lymph node of more than 8 mm on pretreatment imaging.

#### Exclusion criteria

Patients are not eligible for this study if any of the following exclusion criteria apply: rectal tumor > 10 cm from the anal verge on MRI (sagittal slide), cT4 tumor on pretreatment imaging work up (pelvic contrast enhanced MRI and/or endorectal ultrasound) or involvement of external sphincter, circumferential margin ≤2 mm on pretreatment imaging work up (pelvic contrast enhanced MRI), metastatic disease, prior pelvic irradiation or any contraindication to pelvic irradiation, contraindication to oxaliplatin or irinotecan or 5FU based chemotherapy, concomitant treatment with warfarin is contraindicated and warafarin must be replaced whenever possible to allow for inclusion, contraindications to 5-FU: complete and permanent insufficiency in dihydropyrimidine dehydrogenase, bone marrow insufficiency, chronic and severe infection, contraindication to irinotecan: inflammatory bowel disease, bilirubin serum level > 3 times the upper limit of the normal rate, severe bone marrow insufficiency, WHO/ECOG performence status > 2, concomitant treatment with millepertuis, contraindication to oxaliplatin including bone marrow insufficiency before treatment initiation (neutrophil count < 2 × 109/L and/or platelet count < 100 × 109/L), peripheral neuropathy with permanent invalidity before treatment initiation, severe renal insufficiency (Creatinin clearance < 30 ml/min), contraindications to folinic acid including Biermer anemia and other anemia related to B12 vitamin insufficiency, contraindications to capecitabin: severe renal insufficiency (Creatinin clearance < 30 ml/min), live attenuated vaccine should not be used during and 6 months after preoperative treatment, previous colorectal cancer, other concomitant or previous malignancy, except: i/ adequately treated in-situ carcinoma of the uterine cervix, ii/ basal or squamous cell carcinoma of the skin, iii/ cancer in complete remission for > 5 years; presence of any psychological, familial, sociological, or geographical condition potentially hampering compliance with the study protocol and follow-up schedule; those conditions should be discussed with the patient before registration in the trial; protected adults; pregnancy or breastfeeding; patient with no national health or universal plan affiliation coverage.

### Inclusion and randomisation

The overall schedule of each participant in the trial is summarized in supplementary data 2. The eligibility of the patients is confirmed and validated during multidisciplinary meeting including colorectal surgeons, digestive oncologists, radiation oncologists, and radiologists. This multidisciplinary meeting is recommended for any patient with rectal cancer based on French clinical cancer guidelines and is not specific to the study. An inclusion/randomisation visit is organized after the multidisciplinary meeting with a partner physician investigator of the study (surgeon, gastroenterologist, oncologist, or radiotherapist) to inform the patient, to obtain free written informed consent, and to proceed to inclusion and randomization. The randomisation will be made using block of random size and will be stratified according to investigator center and rectal tumour location (< or > 5 cm from the anal verge). The patient is randomized to receive either 6 cycles of modified FOLFIRINOX (experimental arm) or standardized RCT (control arm).

#### Experimental arm

Patients in the experimental arm will receive a modified FOLFIRINOX regimen, one cycle every 14 days as followed Ondansetron 8 mg IV and methylprednisolone 120 mg IV 15 min will be administered before chemotherapy start, Oxaliplatin 85 mg/m^2^ IV 2-h infusion in 250 mL of 5% glucose solution followed by infusion of 50 mL of 5% glucose solution, then Irinotecan 180 mg/m2 IV 90-min infusion in 250 mL of sodium chloride 9 mg/mL (0.9%) or 5% glucose solution, Folinic Acid 400 mg/m^2^ (DL form) or 200 mg/m2 (L form) IV 2-h infusion in 500 mL of 5% glucose solution, along irinotecan infusion, followed by 50 mL 5% glucose solution, then 5-FU 2400 mg/m^2^ given as a continuous infusion over 46 h in 5% glucose solution.

Prophylactic G-CSF administration is recommended when chemotherapy cycle has to be delayed for 1 week or more for neutropenia (see appendix modality of preparation and administration of chemotherapy drugs).

#### Control arm

The RCT protocol is standardized in the trial to limit the variability of tolerance and efficacy of the treatment in the control arm. Radiotherapy will consist in intensity modulation radiation therapy (IMRT) delivering a total dose of 50 Gy in 25 fractions of 2 Gy. Concomitant chemotherapy with capecitabine will be administered from the first to the last day of the radiation treatment (excluding weekends) at a daily dose of 825 mg/m2/12 h. In addition, the study protocol plans a quality control of RCT including a pretest case (benchmark case) at each participating center before the inclusion of the first patient.

### Post treatment MRI reevaluation

The risk of progression of resectable LARC becoming unresectable during systemic modified FOLFIRINOX is low but this theoretical issue cannot be excluded. Besides clinical reevaluation of response and toxicity of preoperative treatment, all patients have MRI reassessment 30 ± 8 days after the end of preoperative treatment in both groups. The aim of this reevaluation is to confirm that CRM remains > 2 mm before surgery to avoid the risk for incomplete (R1) resection. At the time of MRI reassessment, a CRM ≤2 mm is considered as an event of progression for the analysis of the primary endpoint (see definition of progression free survival in “endpoint section” below) and should lead to adapt treatment strategy to avoid loss of chance in the experimental with additional preoperative RCT before surgery.

### Surgery

Surgical resection is performed 6 to 8 weeks after the end of preoperative treatment in both groups. Rectal resection will be performed with respect to French clinical guidelines for oncologic surgery [16]. According to these recommendations, open or laparoscopic proctectomy with total mesorectal excision and > 1 cm distal margin excision should be performed with curative intent. High ligation of inferior mesenteric artery is recommended to obtain adequate lymphadenectomy. Temporary defunctioning ileostomy in patients undergoing sphincter saving surgery and anastomosis is recommended [17].

### Postoperative treatment and follow-up

Indication of postoperative treatment is not standardized in the trial and is left at the discretion of each local multidisciplinary team after discussion on a per-patient basis according to French clinical guidelines [16]. Based on these guidelines, oxaliplatin-based chemotherapy is recommended in ypN+ and R1 resection on final pathologic examination in both groups and postoperative RCT can be proposed as an alternative option in case of R1 resection in the experimental group. In patients with incomplete resection with persistence of macroscopic residual tumoral tissue (R2 resection), postoperative RCT should be proposed in experimental group, and chemotherapy alone in the control group. Post-treatment follow-up is organized with regular clinical and imaging reassessment based on French guidelines. Following completion of study treatment, patients without progression will be followed-up every 3 months during 2 years and thereafter every 6 months for 3 years. In patients who develop disease-progression during the follow up, treatment of the recurrence will be left at physicians discretion based on current French clinical cancer guidelines [18]. In the experimental group, the re use of modified FOLFIRINOX based chemotherapy can be possible and should discussed in patients without residual permanent drug related toxicity.

### Endpoints

The primary endpoint of this phase III study is 3-year progression-free survival (PFS) from the time to randomization. Survival rate will be calculated using the Kaplan-Meier method. In this trial, a modified definition of PFS will be used for the primary endpoint. The rationale for using this modified definition of PFS is to better assess time to failure of the whole treatment strategy (preoperative treatment and surgery).

Progression will be assessed as follows:

- progression during preoperative treatment assessed by MRI reevaluation after chemotherapy or RCT: a circumferential resection margin (CRM) < 2 mm at MRI reevaluation (22–38 days after completion of preoperative treatment) is considered as a progression event. Diagnosis of any new distant lesion whatever the site (liver, lung, adrenal and peritoneum) during preoperative treatment is considered as a progression.
- progression after surgery: PFS will be considered to be the time from randomization to the date of first recurrence/progression after surgery or death, whatever comes first.

Note: Absence of resection or R1 resection per se will not be considered an event for PFS. If a progression is noticed at surgery, it will not count as such as event for PFS (due to the impossibility to determine if the patient has indeed progressed or the disease status was underestimated by initial imaging).

Diagnosis of recurrence or progression can be made only when the clinical and laboratory findings meet at least one of the following criteria objective radiological recurrence or progression on radiological imaging (ultrasound, CT scan, MRI scan, TEP scan as indicated by the clinical picture), positive cytology or biopsy (in case of ascites, anastomotic recurrence, doubt on radiological imaging), death will be considered as an event.

Secondary endpoints include evaluation of treatment related toxicity and evaluation of using the International Common Terminology Criteria for Adverse Events (CTCAE), version 4.0, compliance with study protocol defined as the completion of full-dose preoperative treatment according to the protocol, radiological response on MRI based on tumor size reduction and tumor regression grade (ymrTRG) [19], R0 resection rate (longitudinal and circumferential resection margin > 1 mm) and quality of resection (number of lymph nodes harvested and mesorectal 3 grades Quirke’s grading system), sphincter saving surgery rate, postoperative morbidity and mortality rates, pathologic response after chemotherapy and RCT according to the Rödel Tumor Regression Grading (TRG) system [20], loco-regional recurrence free survival, uncontrolled local recurrence rate, overall survival, bowel function using bowel function dimension of EORTC questionnaire QLQ-CR29 and Low Anterior Resection Syndrome (LARS) score [21] assessed at the inclusion, after preoperative treatment 6 months and 1 year after surgery, sexual function using sexual function dimension of EORTC questionnaire QLQ-CR29 at the inclusion, after preoperative treatment, 6 months and 1 year after surgery, Health-Related Quality of Life (HRQoL) using the French version the European Organization for Research and Treatment of Cancer Quality of Life Questionnaire Core 30 (EORTC QLQ-C30) version 3.0, with the specific CRC module (QLQ-CR29) [22, 23] at the inclusion, after preoperative treatment, months and 1 year after surgery. All the study data will be collected in an electronic clinical research form (eCRF Cleanweb®) that has been designed and validated by the steer committee and that will be monitored by the Unité de Recherche Clinique Fernand Widal – Lariboisière. All adverse events are also collected in the eCRF and each adverse events will graded according to its severity using the CTCAE v4. For each adverse event, the investigator must assess the causal relationship of between protocol treatment (chemotherapy, chemoradiotherapy, surgery) and the occurrence of the event. The investigators have to declare systematically serious adverse events to the sponsor.

### Statistical analysis

A sample size of 551 patients, based on an expected accrual duration of 36 months, 60 months minimum follow-up, and an expected 3 year DFS rate in the preoperative RCT arm of 70%, is expected to provide 295 DFS events required to provide 80% power to declare non-inferiority of the preoperative chemotherapy arm when the true hazard ratio between arms is 1.0 (H1). This design has a type one-error rate of 0.05 if the true hazard ratio between arms is 1.34 (H0). This hazard rate, in an exponential survival model, corresponds to a decrease in the 3-year DFS rate on the preoperative chemotherapy arm to 62%. In this design the critical driver of the analysis timing is the number of DFS events, as the precise accrual and follow-up patterns will differ by study and are impossible to precisely anticipate. Two interim analyses for efficacy and futility for the primary end point were planned and will be conducted at 0.11 and 0.33 (approximately 33 and 98 DFS events) information fraction using an O’Brien-Fleming stopping boundary. By considering a rate of 4% for not informative or lost to follow-up patients the total number of patients to be included in this trial was 551*100/96 = 574 patients. The access to study data and all statistical analyses is under the responsibility of the Unité de Recherche Clinique Fernand Widal – Lariboisière (EV).

### Ethics and safety

This study (version 1.1) has been approved by a national Institutional Review Board: the Regional Comity of Patients Protection of Ile de France VI, CPP 77–18, Dossier n° 18.10.18.66606. All protocol modifications have first to be declared and validated by a new CPP assessment. The institutional promoter is the Assistance Publique Hôpitaux de Paris, France. The trial has been registered on ClinicalTrials.gov website under the identification number NCT03875781 on March 20,197. This study received a grant from the Ministry of Social Affairs and Health of France (PHRC-K 2017). The study complies with the Declaration of Helsinki rules and the principles of the Good Clinical Practices guidelines.

## Discussion

### The rationale of omitting pelvic irradiation in a CRM based strategy to locally advanced rectal cancer

Despite its interest in improving local control of rectal cancer patients, preoperative RCT has also several disadvantages. First, preoperative RCT has never been shown to decrease the risk of metastatic recurrence and to improve survival in rectal cancer patients undergoing total mesorectal excision regardless pathological tumor stage [3, 10]. Second, RCT is associated with an increased risk of late side effects on intestinal and genitourinary functions. Compared with patients undergoing upfront surgery, patients who have been treated with preoperative pelvic irradiation have an increased risk of poor intestinal functional results. Risk of occurrence of some degree of fecal incontinence can be up to 62% in patients undergoing rectal surgery after RCT compared to 38% in patients undergoing upfront surgery [24, 25]. The risk of severe low anterior resection syndrome (LARS) is also significantly increased in patients treated with preoperative pelvic irradiation, with an incidence of severe LARS of 56% after irradiation compared to 35% after surgery alone [12]. Preoperative pelvic irradiation is also associated with an increased risk of impaired sexual functioning both in males and females [11, 26]. Finally, some dimensions of quality of life including physical functioning can be deteriorated in patients treated with preoperative RCT [11, 26]. The absence of proven benefit on long-term survival and the risk of late side effects associated with RCT questions its routine use for all T3 or T1-T3N+ rectal cancer patients.

### The choice of modified FOLFIRINOX as preoperative treatment for locally advanced rectal cancer

Recent phase II and one phase III studies evaluated the feasibility and the safety of preoperative oxaliplatin-based chemotherapy without pelvic irradiation prior to total mesorectal excision in selected patients with cT2-T3Nx rectal cancer [13–15, 27]. These studies have shown that preoperative chemotherapy alone was associated with a high rate of complete R0 resection (100%), a low risk of postoperative local recurrence (0–2%) and progression free-survival up to 92% at 4-year in selected LARC patients. Triplet combination regimen appears to be particularly effective in rectal cancer and several recent phase II studies have used modified FOLFIRINOX either as induction chemotherapy in a tailored approach to LARC [28] or in metastatic rectal cancer [29]. GRECCAR 4 phase II trial reported that a 80% objective response rate (more than 50% size reduction of tumor) of patients with LARC [28] after four cycles. In the FFCD 1102 phase II trial, 90% of patients with metastatic rectal cancer had adequate control of symptoms of the primary tumor and 55% of patients had major radiologic response (defined by tumor volume reduction > 70%) on MRI in primary tumor after eight cycles [29]. At this time, long-term data comparing systemic chemotherapy using modified FOLFIRINOX and standard RCT have not been reported. The demonstration of the non-inferiority of preoperative chemotherapy alone compared to RCT in locally advanced resectable rectal cancer on oncologic outcomes may lead to avoid late side effects on functional results associated with pelvic irradiation without increased risks of locoregional recurrence. In addition, full dose chemotherapy before surgery may have a beneficial effect on long-term oncologic outcome as a substantial number of patients do not receive postoperative chemotherapy after total mesorectal excision following preoperative radiotherapy because of postoperative complications [30, 31]. The validation of this new therapeutic strategy is therefore of major interest for rectal cancer patients and could lead to a change to standard treatment to these patients.

### The choice of the study design and primary outcome measure

The administration of preoperative chemotherapy without pelvic irradiation may have substantial advantages compared to standard RCT especially in cancer survivors by decreasing the risk of late side effects and the negative impact of treatment on long term quality of life. This approach has first to be demonstrated as safe and at least non inferior to the standard treatment in term of oncologic outcome. The interest of preoperative RCT has been demonstrated on the reduction of the risk of local recurrence. However, this criterion is not adequate to objectively evaluate the quality of the whole treatment strategy. The last important trials evaluating new treatment modalities in rectal cancer have rather used disease-free or progression free survival [5, 6] which is a strong surrogate marker of overall survival in cancer patients [32]. Of course, the study aims at demonstrating the non inferiority of preoperative chemotherapy using modified FOLFIRINOX in terms of progression free survival compared to RCT; the second objective is to show that it may limit the risk of long term side effects of treatment. This non inferiority phase III design has been discussed and validated in 3 French surgical and cancer societies, namely FRENCH, GRECCAR, and PRODIGE. The implication of these groups will favor and guarantee the capacities of inclusion of the trial. If the trial is positive, it may lead to an important change in the treatment strategy to patients with resectable LARC.

## Supplementary information

**Additional file 1.** List of participating centers.

**Additional file 2.** The overall schedule of each participant in the trial.

## Data Availability

The study protocol is available on the website clinicaltrials.gov at the url: https://clinicaltrials.gov/ct2/show/NCT03875781 All data regarding ethical aspects are available at the Unité de recherché Clinique – Lariboisière – Assistance Publique Hôpitaux de Paris.

## Abbreviations

RCT: radiochemotherapy
LARC: locally advanced rectal cancer
CRM: circumferential resection margin

## References

[1] Heald RJ, Ryall RD (1986). Recurrence and survival after total mesorectal excision for rectal cancer. Lancet..

[2] Bosset J-F, Collette L, Calais G, Mineur L, Maingon P, Radosevic-Jelic L (2006). Chemotherapy with preoperative radiotherapy in rectal cancer. N Engl J Med.

[3] Kapiteijn E, Marijnen CA, Nagtegaal ID, Putter H, Steup WH, Wiggers T (2001). Preoperative radiotherapy combined with total mesorectal excision for resectable rectal cancer. N Engl J Med.

[4] Gérard J-P, Conroy T, Bonnetain F, Bouché O, Chapet O, Closon-Dejardin M-T (2006). Preoperative radiotherapy with or without concurrent fluorouracil and leucovorin in T3-4 rectal cancers: results of FFCD 9203. J Clin Oncol.

[5] Rödel C, Graeven U, Fietkau R, Hohenberger W, Hothorn T, Arnold D (2015). Oxaliplatin added to fluorouracil-based preoperative chemoradiotherapy and postoperative chemotherapy of locally advanced rectal cancer (the German CAO/ARO/AIO-04 study): final results of the multicentre, open-label, randomised, phase 3 trial. Lancet Oncol..

[6] Gérard J-P, Azria D, Gourgou-Bourgade S, Martel-Lafay I, Hennequin C, Etienne P-L (2012). Clinical outcome of the ACCORD 12/0405 PRODIGE 2 randomized trial in rectal cancer. J Clin Oncol.

[7] Beets-Tan RG, Beets GL, Vliegen RF, Kessels AG, Van Boven H, De Bruine A (2001). Accuracy of magnetic resonance imaging in prediction of tumour-free resection margin in rectal cancer surgery. Lancet..

[8] Op de Beeck B, Smeets P, Penninckx F, Pattyn P, Silversmit G, Van Eycken E (2017). Accuracy of pre-treatment locoregional rectal cancer staging in a national improvement project. Acta Chir Belg..

[9] Taylor FGM, Quirke P, Heald RJ, Moran B, Blomqvist L, Swift I (2011). Preoperative high-resolution magnetic resonance imaging can identify good prognosis stage I, II, and III rectal cancer best managed by surgery alone: a prospective, multicenter. European study Ann Surg.

[10] van Gijn W, Marijnen CAM, Nagtegaal ID, Kranenbarg EM-K, Putter H, Wiggers T (2011). Preoperative radiotherapy combined with total mesorectal excision for resectable rectal cancer: 12-year follow-up of the multicentre, randomised controlled TME trial. Lancet Oncol..

[11] Wiltink LM, Marijnen CAM, Meershoek-Klein Kranenbarg E, van de Velde CJH, Nout RA (2016). A comprehensive longitudinal overview of health-related quality of life and symptoms after treatment for rectal cancer in the TME trial. Acta Oncol.

[12] Chen TY-T, Wiltink LM, Nout RA, Meershoek-Klein Kranenbarg E, Laurberg S, Marijnen CAM (2015). Bowel function 14 years after preoperative short-course radiotherapy and total mesorectal excision for rectal cancer: report of a multicenter randomized trial. Clin Colorectal Cancer.

[13] Schrag D, Weiser MR, Goodman KA, Gonen M, Hollywood E, Cercek A (2014). Neoadjuvant chemotherapy without routine use of radiation therapy for patients with locally advanced rectal cancer: a pilot trial. J Clin Oncol.

[14] Fernandez-Martos C, Brown G, Estevan R, Salud A, Montagut C, Maurel J (2014). Preoperative chemotherapy in patients with intermediate-risk rectal adenocarcinoma selected by high-resolution magnetic resonance imaging: the GEMCAD 0801 phase II multicenter trial. Oncologist..

[15] Bensignor T, Brouquet A, Dariane C, Thirot-Bidault A, Lazure T, Julié C (2015). Pathological response of locally advanced rectal cancer to preoperative chemotherapy without pelvic irradiation. Color Dis.

[16] Slim K, Blay JY, Brouquet A, Chatelain D, Comy M, Delpero JR (2009). Digestive oncology: surgical practices. J Chir (Paris).

[17] Matthiessen P, Hallböök O, Rutegård J, Simert G, Sjödahl R (2007). Defunctioning stoma reduces symptomatic anastomotic leakage after low anterior resection of the rectum for cancer: a randomized multicenter trial. Ann Surg.

[18] Gérard J-P, André T, Bibeau F, Conroy T, Legoux J-L, Portier G (2017). Rectal cancer: French intergroup clinical practice guidelines for diagnosis, treatments and follow-up (SNFGE, FFCD, GERCOR, UNICANCER, SFCD, SFED, SFRO). Dig Liver Dis.

[19] Patel UB, Brown G, Rutten H, West N, Sebag-Montefiore D, Glynne-Jones R (2012). Comparison of magnetic resonance imaging and histopathological response to chemoradiotherapy in locally advanced rectal cancer. Ann Surg Oncol.

[20] Rödel C, Martus P, Papadoupolos T, Füzesi L, Klimpfinger M, Fietkau R (2005). Prognostic significance of tumor regression after preoperative chemoradiotherapy for rectal cancer. J Clin Oncol.

[21] Juul T, Ahlberg M, Biondo S, Emmertsen KJ, Espin E, Jimenez LM (2014). International validation of the low anterior resection syndrome score. Ann Surg.

[22] Sprangers MA, Cull A, Bjordal K, Groenvold M, Aaronson NK (1993). The European Organization for Research and Treatment of Cancer. Approach to quality of life assessment: guidelines for developing questionnaire modules. EORTC study group on quality of life. Qual Life Res.

[23] Whistance RN, Conroy T, Chie W, Costantini A, Sezer O, Koller M (2009). Clinical and psychometric validation of the EORTC QLQ-CR29 questionnaire module to assess health-related quality of life in patients with colorectal cancer. Eur J Cancer.

[24] Parc Y, Zutshi M, Zalinski S, Ruppert R, Fürst A, Fazio VW (2009). Preoperative radiotherapy is associated with worse functional results after coloanal anastomosis for rectal cancer. Dis Colon Rectum.

[25] Peeters KCMJ, van de Velde CJH, Leer JWH, Martijn H, Junggeburt JMC, Kranenbarg EK (2005). Late side effects of short-course preoperative radiotherapy combined with total mesorectal excision for rectal cancer: increased bowel dysfunction in irradiated patients--a Dutch colorectal cancer group study. J Clin Oncol.

[26] Marijnen CAM, van de Velde CJH, Putter H, van den Brink M, Maas CP, Martijn H (2005). Impact of short-term preoperative radiotherapy on health-related quality of life and sexual functioning in primary rectal cancer: report of a multicenter randomized trial. J Clin Oncol.

[27] Deng Y, Chi P, Lan P, Wang L, Chen W, Cui L (2016). Modified FOLFOX6 with or without radiation versus fluorouracil and Leucovorin with radiation in Neoadjuvant treatment of locally advanced rectal Cancer: initial results of the Chinese FOWARC multicenter, open-label, randomized three-arm phase III trial. J Clin Oncol.

[28] Rouanet P, Rullier E, Lelong B, Maingon P, Tuech J-J, Pezet D (2017). Tailored treatment strategy for locally advanced rectal carcinoma based on the tumor response to induction chemotherapy: preliminary results of the French phase II multicenter GRECCAR4 trial. Dis Colon Rectum.

[29] Bachet JB, Lucidarme O, Levache CB, Barbier E, Raoul JL, Lecomte T (2018). FOLFIRINOX as induction treatment in rectal cancer patients with synchronous metastases: results of the FFCD 1102 phase II trial. Eur J Cancer.

[30] Bujko K, Glimelius B, Valentini V, Michalski W, Spalek M (2015). Postoperative chemotherapy in patients with rectal cancer receiving preoperative radio(chemo)therapy: a meta-analysis of randomized trials comparing surgery ± a fluoropyrimidine and surgery + a fluoropyrimidine ± oxaliplatin. Eur J Surg Oncol.

[31] Breugom AJ, Swets M, Bosset J-F, Collette L, Sainato A, Cionini L (2015). Adjuvant chemotherapy after preoperative (chemo)radiotherapy and surgery for patients with rectal cancer: a systematic review and meta-analysis of individual patient data. Lancet Oncol.

[32] Buyse M, Burzykowski T, Carroll K, Michiels S, Sargent DJ, Miller LL (2007). Progression-free survival is a surrogate for survival in advanced colorectal cancer. J Clin Oncol.

