# Supplementary material for: NORAD01-GRECCAR16 multicenter phase III non-inferiority randomized trial comparing preoperative modified FOLFIRINOX without irradiation to radiochemotherapy for resectable locally advanced rectal cancer (intergroup FRENCH-GRECCAR- PRODIGE trial)
Source: BMC Cancer. 2020 May 29;20:485. doi: 10.1186/s12885-020-06968-1 (PMC7257230; doi:10.1186/s12885-020-06968-1)
Supplement: Supplementary file 1 — Additional file 1. List of participating centers. [file 12885_2020_6968_MOESM1_ESM.docx]

Supplementary data 1 : list of participating centers

| *Team* | *Name* | *Specialty* | *Health institution* |
| --- | --- | --- | --- |
| *Team 1 (coordination team)* | *Coordinator*  **Benoist Stéphane** | *Oncologic and digestive surgery* | APHP, Bicêtre hospital |
| *Team 2* | ***Jean-Baptiste Bachet*** | *Digestive oncology* | APHP, Pitié Salpétrière Hospital |
| *Team 3* | *Investigator*  **Florence Huguet** | *Radiation Oncology* | APHP, Tenon Hospital |
| *Team 4* | *Head*  **Jérémie Lefèvre** | *General and digestive surgery* | APHP, Saint Antoine Hospital |
| *Team 5* | *Head*  **Guillaume Piessen** | *Oncologic and digestive surgery* | Lille University Hospital |
| *Team 6* | *Head*  **Philippe Rouanet** | *Surgical Oncology* | CLCC Val d’Aurelle |
| *Team 7* | *Head*  **Frederic Dumont** | *Surgical Oncology* | CLCC Nantes |
| *Team 8* | *Head*  **Zaher Lakkis** | *Oncologic and digestive surgery* | Besancon University Hospital |
| *Team 9* | *Head*  **Mehdi Ouaissi** | *General and digestive surgery* | Tours University Hospital |
| *Team 10* | *Head*  **Laura Beyer-Berjot** | *General and digestive surgery* | Marseille Nord University Hospital |
| *Team 11* | *Head*  **Laurent mineur** | *radiation oncology* | St Catherine Institut |
| *Team 12* | *Head*  **Adeline Germain** | *Digestive surgery* | Nancy University hospital |
| *Team 13* | *Head*  **Frédérique Peschaud** | *Oncologic and digestive surgery* | APHP, Ambroise Paré hospital |
| *Team 14* | *Head*  **Quentin Denost** | *Colorectal surgery* | Bordeaux University Hospital |
| *Team 15* | *Head*  **Jerome Loriau** | *Digestive surgery* | Saint Joseph Hospital, Paris |
| *Team 16* | *Head*  **Bernard Meunier** | *Digestive surgery* | Rennes university hospital |
| *Team 17* | *Head*  **Jean-Jacques Tuech** | *Digestive surgery* | Rouen university hospital |
| *Team 18* | *Head*  **Eddy Cotte** | *Oncologic and digestive surgery* | Lyon Sud University Hospital |
| *Team 19* | *Head*  **Anne Dubois** | *Digestive surgery* | Clermont University Hospital |
| *Team 20* | *Head*  **Jean-Luc Faucheron** | *Digestive surgery* | Grenoble University Hospital |
| *Team 21* | *Head*  **Bertrand Dousset** | *Digestive surgery* | APHP, Cochin Hospital |
| *Team 22* | *Head*  **Jean Marc Phelip** | *Digestive Oncology* | CHU Saint Etienne |
| *Team 23* | *Head*  **Serge Evrard** | *Surgical Oncology* | CLCC Bordeaux |
| *Team 24* | *Head*  **Léon Maggiori** | *Colorectal surgery* | APHP, Beaujon Hospital |
| *Team 25* | *Head*  **Eric Francois** | *Digestive oncology* | CLCC Nice |
| *Team 26* | *Head*  **Jean-Marc Regimbeau** | *Digestive surgery* | Amiens University Hospital |
| *Team 27* | *Head*  **Muriel Mathonnet** | *Digestive surgery* | Limoges university hospital |
| *Team 28* | *Head*  **Leonor benhaim** | *Oncologic and digestive surgery* | CLCC Gustave Roussy Villejuif |
| *Team 29* | *Head*  **Sylvain Manfredi** | *Digestive oncology* | Dijon University Hospital |
| *Team 30* | *Head*  **Christine Denet** | *Digestive surgery* | Montsouris Institute, Paris |
| *Team 31* | *Head*  **Arnaud Alves** | *Digestive surgery* | Caen University Hospital |
| *Team 32* | *Head*  **Pascal Artru** | *Digestive oncology* | Hopital Prive Jean Mermoz, Lyon |
| *Team 33* | *Head*  **Hadrien Tranchart** | *Digestive surgery* | APHP beclere Hospital |
| *Team 34* | *Head*  **Sophie Deguelte Lardiere** | *Digestive surgery* | Reims University Hospital |
| *Team 35* | *Head*  **Diane Goere** | *Digestive surgery* | APHP, Saint Louis Hospital |
| *Team 36* | *Head*  **Francois Mauvais** | *Digestive surgery* | Beauvais Hospital |
| *Team 37* | *Head*  **Michel Rivoire** | *Oncologic and digestive surgery* | CLCC Léon Bérard, Lyon |
| *Team 38* | *Head*  **Julien Taieb** | *Digestive Oncology* | APHP, HEGP |
| *Team 39* | *Head*  **Benoît Romain** | Digestive Surgery | Hôpital Hautepierre  Strasbourg |
| *Team 40* | *Head*  **Bernard Lelong** | *Oncologic and digestive surgery* | CLCC Institut Paoli Calmettes |
