# Supplementary material for: NORAD01-GRECCAR16 multicenter phase III non-inferiority randomized trial comparing preoperative modified FOLFIRINOX without irradiation to radiochemotherapy for resectable locally advanced rectal cancer (intergroup FRENCH-GRECCAR- PRODIGE trial)
Source: BMC Cancer. 2020 May 29;20:485. doi: 10.1186/s12885-020-06968-1 (PMC7257230; doi:10.1186/s12885-020-06968-1)
Supplement: Supplementary file 2 — Additional file 2. The overall schedule of each participant in the trial. [file 12885_2020_6968_MOESM2_ESM.docx]

Supplementary data 2: The overall schedule of each participant in the trial

|  | **Screening / Baseline** | | **RANDOMIZATION** | **Preoperative treatment** | | | | **Preoperative assessment** | | **SURGERY** | **Post-surgery** | | **Postoperative treatment^b^** | | | | **Follow-up** | |
| --- | --- | --- | --- | --- | --- | --- | --- | --- | --- | --- | --- | --- | --- | --- | --- | --- | --- | --- |
|  | **Within 30 days before randomisation** | |  | **Before each cycle** | | **At the end of each cycle** | | **30±8 days after preoperative treatment** | |  | **4 weeks after surgery** | | **Before each cycle** | | **At the end of each cycle** | | **Every 3 months for**  **2 years; then every**  **6 months for 3 years** | |
|  | **C** | **R** |  | **C** | **R** | **C** | **R** | **C** | **R** |  | **C** | **R** | **C** | **R** | **C** | **R** | **C** | **R** |
| Checking inclusion / non inclusion criteria |  | **X** |  |  |  |  |  |  |  |  |  |  |  |  |  |  |  |  |
| Informed consent(s) |  | **X** |  |  |  |  |  |  |  |  |  |  |  |  |  |  |  |  |
| Demographic data | **X** |  |  |  |  |  |  |  |  |  |  |  |  |  |  |  |  |  |
| Medical history | **X** |  |  |  |  |  |  |  |  |  |  |  |  |  |  |  |  |  |
| Previous CRC treatment history | **X** |  |  |  |  |  |  |  |  |  |  |  |  |  |  |  |  |  |
| Physical examination | **X** |  |  | **X** |  |  |  | **X** |  |  | **X** |  | **X** |  |  |  | **X** |  |
| WHO/ECOG performance status | **X** |  |  | **X** |  |  |  | **X** |  |  | **X** |  | **X** |  |  |  | **X** |  |
| electrocardiogram | **X** |  |  |  |  |  |  | **X** |  |  | **X** |  |  |  |  |  |  |  |
| Assessment of digestive symptoms | **X** |  |  | **X** |  |  |  | **X** |  |  | **X** |  | **X** |  |  |  |  |  |
| Complete study of the colon and the rectum | **X** |  |  |  |  |  |  |  |  |  |  |  |  |  |  |  |  |  |
| Rectal tumor location from the anal verge | **X** |  |  |  |  |  |  |  |  |  |  |  |  |  |  |  |  |  |
| Biopsy of rectal tumor | **X** |  |  |  |  |  |  |  |  |  |  |  |  |  |  |  |  |  |
| Absence of synchronous colorectal cancer or polyposis | **X** |  |  |  |  |  |  |  |  |  |  |  |  |  |  |  |  |  |
| Rectal MRI assessment | **X** |  |  |  |  |  |  |  | **X** |  |  |  |  |  |  |  |  |  |
| CT scan (abdominal, pelvis and chest) +IV contrast | **X** |  |  |  |  |  |  | **X^a^** |  |  |  |  |  |  |  |  | **X** |  |
| Blood sample tests for hematology (hemoglobin, leucocyts and platelets count) and biochemistry (Na, K, Ph, creatinine, Ca, Ph, Mg and albumin) | **X** |  |  | **X** |  | **X** |  |  |  |  |  |  | **X** |  | **X** |  |  |  |
| Liver function tests: Alkaline phosphatase, ASAT and ALAT, Total bilirubin, γGT | **X** |  |  | **X** |  | **X** |  |  |  |  |  |  | **X** |  | **X** |  |  |  |
| Prothrombin time | **X** |  |  |  |  |  |  | **X** |  |  |  |  |  |  |  |  |  |  |
| CEA,  CA19-9 (optional) | **X** |  |  |  |  |  |  | **X** |  |  |  |  |  |  |  |  | **X** |  |
| Serum pregnancy test (for women of childbearing potential) within 14 days before randomization | **X** |  |  |  |  |  |  |  |  |  |  |  |  |  |  |  |  |  |
| Toxicity and adverse events |  |  |  |  |  | **X** |  | **X** |  |  | **X** |  |  |  | **X** |  |  |  |
| EORTC QLQ-C30 |  | **X** |  |  |  |  |  |  | **X** |  |  |  |  |  |  |  |  | **X** |
| EORTC QLQ-CR29 |  | **X** |  |  |  |  |  |  | **X** |  |  |  |  |  |  |  |  | **X** |
| LARS score |  | **X** |  |  |  |  |  |  | **X** |  |  |  |  |  |  |  |  | **X** |
| Preanesthesic assesment |  | **X** |  |  |  |  |  | **X** |  |  |  | **X** |  |  |  |  |  | **X** |
| Translational research collection^b^ |  | **X** |  |  |  |  |  |  | **X** |  |  | **X** |  |  |  |  |  | **X** |
